# The Edge Application of Machine Learning Techniques for Fault Diagnosis in Electrical Machines

**DOI:** 10.3390/s23052649

**Published:** 2023-02-28

**Authors:** Javier de las Morenas, Francisco Moya-Fernández, Julio Alberto López-Gómez

**Affiliations:** 1 Mining and Industrial Engineering School of Almadén, University of Castilla-La Mancha, 13400 Almadén, Spain; 2 Mantis Research Group, EIIA Toledo, University of Castilla-La Mancha, 45005 Toledo, Spain

**Keywords:** fault diagnosis, edge computing, machine learning, motor current signature analysis

## Abstract

The advent of digitization has brought about new technologies that enable advanced condition monitoring and fault diagnosis under the Industry 4.0 paradigm. While vibration signal analysis is a commonly used method for fault detection in literature, it often involves the use of expensive equipment in difficult-to-reach locations. This paper presents a solution for fault diagnosis of electrical machines by utilizing machine learning techniques on the edge, classifying information coming from motor current signature analysis (MCSA) for broken rotor bar detection. The paper covers the process of feature extraction, classification, and model training and testing for three different machine learning methods using a public dataset to then export the results to diagnose a different machine. An edge computing approach is adopted for the data acquisition, signal processing and model implementation on an affordable platform, the Arduino. This makes it accessible for small and medium-sized companies, albeit with the limitations of a resource-constrained platform. The proposed solution has been tested on electrical machines in the Mining and Industrial Engineering School of Almadén (UCLM) with positive results.

## 1. Introduction

Society is currently deeply immersed in the fourth industrial revolution, where digitalization has led to the emergence of new technologies and lines of research, such as the Internet of Things, Big Data, Smart technology, and Cloud Computing, all with the goal of digitalizing information.

The digitalization process is having a significant impact not only in industry but also in other areas, such as smart cities and buildings [1], energy systems [2], pollution monitoring [3], health tracking [4], and intelligent transportation systems [5]. However, its full realization within industry has yet to be fully achieved, and its impact on small and medium-sized enterprises (SMEs) is considerably lower.

In Spain, Industry 4.0 is known as Connected Industry 4.0 [6]. In order to assist SMEs in the development of digitalization, the government has created the Digital Transformation Offices within red.es (a public entity attached to the Ministry of Economic Affairs and Digital Transformation) [7]. This is not insignificant, as SMEs make up 99.6% of total companies in Spain according to data from the Social Welfare, as of December 2022, including 59.7% of the total number of workers [8].

The present paper is focused on one of the fundamental concepts of Industry 4.0, namely predictive maintenance, with a specific emphasis on fault diagnosis in electrical machines. As per the International Energy Agency report [9], approximately 70% of all energy expended in industries in developed countries is consumed by electric motors. Therefore, there has been a significant increase in interest in predicting and detecting faults in these machines [10,11,12,13], and the advent of Industry 4.0 provides an additional boost for this trend [14].

Induction motors are protected by devices such as relays, circuit breakers, etc., but the performance of these protections is limited either to protecting the motor against an external problem or to a significant level of motor deterioration when the fault is often irreversible. On the other hand, the demand from users to increase the reliability of electric motors is constantly growing due to the importance of many applications, advances in technology and the need to obtain better operating results. Therefore, the ability to predict and detect the onset of failures becomes crucial in applications where the complexity, criticality, cost of shutdown or repair, and safety considerations do not permit unexpected motor failure [15].

This paper presents a solution for the fault diagnosis of electrical machines in an edge computing approach. The novelty of this work is the constant condition monitoring of the machines and the edge application of machine learning classifying methods based on a low-cost and resource-constrained platform, with the objective of reducing maintenance costs and equipment downtime through the detection of equipment faults, from the perspective of making it accessible for SMEs. Another innovative aspect is the extrapolation of the classification model. This model has been developed using a dataset from a specific machine, and it is now being applied to other machines. This process requires the selection of features that are independent of the absolute magnitudes of each motor. Novel features have been proposed.

The subsequent sections of this paper are structured as follows: In Section 2, the prevalent methods for fault diagnosis in electric induction motors are discussed, along with a brief overview of Machine Learning techniques and Industrial Cyber-Physical Systems (ICPS). Section 3 presents the components of the ICPS system responsible for fault diagnosis and emphasizes their implementation on platforms with limited resources using an edge computing approach, which enables local data processing. Section 4 presents the public dataset used, the development of the classification models, the practical implementation of the proposed solution, and the detailed analysis of the results. Lastly, Section 5 concludes the paper by summarizing our findings and outlining potential future avenues for research.

## 2. State of the Art

Several diagnostic techniques have been established for three-phase induction motors. These techniques are primarily based on the examination of the magnitudes that are accessible at the motor terminals or those faults that can be detected through appropriate equipment by performing measurements under normal operating conditions of the motor or when the machine is out of service.

### 2.1. Out-of-Service Diagnostics

The techniques for diagnosing three-phase induction motors typically involve conducting evaluations when the machine is not in operation. This often requires the motor to be shut off and, in some cases, partially or fully disassembled. These evaluations are typically performed during scheduled maintenance intervals. The majority of these methods focus on assessing the condition of the insulation system and the state of the magnetic core. The methods utilized for the diagnosis of three-phase induction motors include, but are not limited to [16]:

- Measurement of insulation resistance to ground, including the determination of polarization index, absorption current, tangent delta capacitance, loss factor, and overvoltage tests.
- Analysis of insulation between turns, utilizing techniques such as the shock wave test, measurement of insulation resistance, reverse sequence impedance, electrical circuit analysis, and parameter estimation.
- Examination of the magnetic core through the application of nominal or reduced flux.
- Analysis of bearing lubricants, including the examination of physical characteristics and particle content.

### 2.2. In-Service Diagnostics

In-service diagnostic methods offer the advantage of providing information on the state of the machine without interrupting its operation. This is a significant advantage, as these methods typically include a series of analysis utilities, commonly in the form of software, which can be utilized in predictive maintenance programs, making their implementation highly desirable.

The most prominent methods for detecting faults through Condition Monitoring (CM) are [10]:

- Stator current analysis or motor current signature analysis (MCSA) [17,18].
- Vibration analysis [19,20,21].
- Temperature measurement (thermography) [22,23,24].
- Partial discharges.
- Gas analysis.
- Axial dispersion flux analysis.
- Torque and speed fluctuations.
- Input power analysis.

Motor Current Signature Analysis (MCSA) is a non-invasive diagnostic technique used to analyze the health of induction motors. It involves analyzing the current waveform generated by the motor during normal operation and comparing it to a known good signature to detect anomalies and faults. It represents the first step in fault diagnosis, typically accompanied by signal processing techniques such as Fast Fourier Transform (FFT) [19,25], wavelet [17,26] or Hilbert–Huang Transform [27,28]. MCSA can provide inaccurate results with the occurrence of saturation, interbar currents or magnetic asymmetry [29].

Reference [17] studied two techniques of signal processing to diagnose inter-turn short circuit (ITSC) and the unbalanced voltage supply (UVS) using MCSA and processing the information first with Fast Fourier Transform (FFT) and then with Discrete Wavelet Energy Ratio (DWER). Reference [18] used MCSA for detecting the combination of bearing faults by applying Discrete wavelet transform (DWT) to de-noise the signal, and then a pre-fault component cancellation using an adaptive filter (Wiener filter) to finally estimate the fault using Matrix Pencil method (MPM). A similar approach with MCSA and Wiener filter is taken in [30] for several types of fault detection. In [31] is presented a guideline for avoiding false fault detection based on MCSA. The guideline includes studying the relationship between other faults and the potential for misidentification, as well as strategies for overcoming such issues. MCSA is mainly applied in stationary conditions, but it is also interesting to analyze nonstationary conditions as in [32], which is focused on detecting eccentricities in the startup time modeling the induction motor, or in [27], which uses new transient-based diagnosis approaches involving time-frequency transformations.

There are alternatives to the application of MCSA, mainly the analysis of vibration signals. Accelerometers are used for the acquisition of vibration data, and their adequate location conveys a great part of the result. Reference [19] proposed to utilize vibration signals for detecting faults in rotors, with the support of classification machine learning techniques. It is typical to utilize FFT in vibration data signal processing [33]. The fracture of races in bearings can be detected by utilizing radial and axial accelerometers [20]. In the event of bearing faults or imbalance, it is possible to measure the vibration of the machine through an antenna and subsequently classify the issue using deep learning techniques [34]. The combined usage of vibration and stator current signals shows great promise in accurately classifying broken rotor bars [21].

Thermography is a method commonly used for diagnosing faults in machinery. To effectively apply this technique, it is necessary firstly to pre-process the images, for example using the region of interest (ROI). Once this has been done, the images can then be classified using machine learning algorithms [23]. It is a common method in short-circuit faults or inter-turn faults [24]. Model-based diagnosis is another alternative for fault diagnosis in electrical machines [11,35].

In this paper, MCSA is selected as a non-invasive, cost-effective, and reliable technology [12,36]. The next subsections briefly present some faults detected with Motor Current Signature Analysis.

#### 2.2.1. Eccentricity Faults

**Figure 1:**
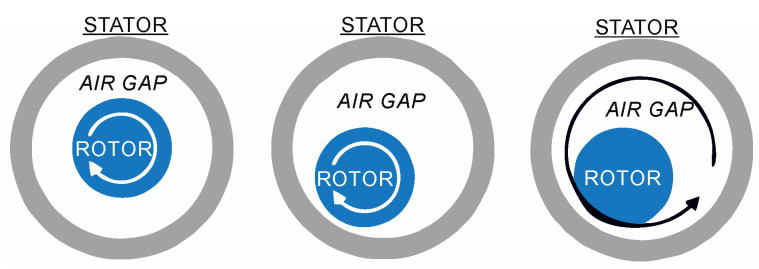
Healthy motor (**left**), static eccentricity fault (**center**), and dynamic eccentricity (**right**).

Two distinct types of eccentricity faults can occur: static and dynamic. Figure 1 illustrates both.

Eccentricity is related to air gap distortion. When a mixed eccentricity fault occurs, sidebands of the fundamental frequency of the power supply appear. These sideband frequencies are given by [10,11,32]:

(1) $$f_{ecc}=f_{s}\left[1\pm m\left(\frac{1-s}{p}\right)\right]$$

where *f_s_* is the supply frequency, *s* is the slip, *p* is the number of pole pairs, and *m* = 1, 2, 3, …. In contrast to other approaches, this method does not necessitate knowledge of the mechanical properties of the motor.

#### 2.2.2. Broken Rotor Bars

**Figure 2:**
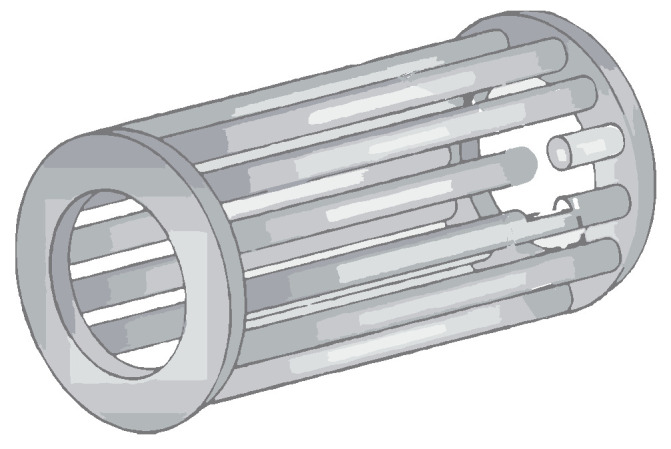
Broken rotor bar.

The breaking of rotor bars is one of the primary causes of failure in induction motors, particularly in high-power motors that are frequently started under load. Figure 2 shows an example of a broken rotor bar.

The presence of broken rotor bars in a motor results in the destruction of rotor symmetry, which, in turn, generates a rotating field that produces harmonics in the stator current as described by [36,37]:

(2) $$f_{brb2}=f_{s}\left[1\pm k2s\right]$$

where *k* = 1, 2, 3, 4, ….

#### 2.2.3. Bearing Damage

**Figure 3:**
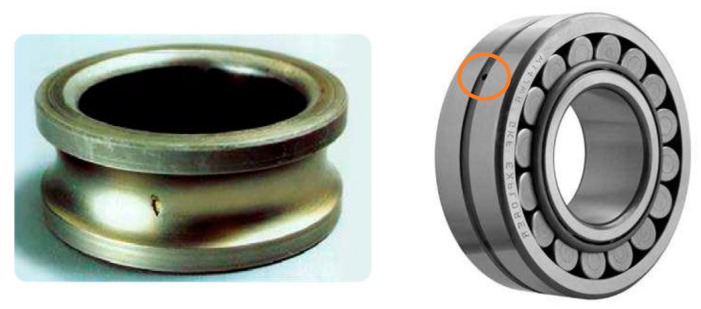
Bearing damage, inner ring (**left**) and outer ring (**right**) [38].

The prevalent cause of installation issues is the improper application of force when installing the bearing onto the shaft or within the housing. This can result in physical damage, such as brinelling or false brinelling of the raceways, leading to an early failure of the bearing. Figure 3 shows two examples of bearing damage.

The mechanical displacement caused by damaged bearings leads to variations in the machine air gap, which can be characterized by a combination of rotating eccentricities that move in both directions. Similar to the deviation in the air gap, these fluctuations generate stator currents at specific frequencies [10,35,37]:

(3) $$f_{bng}=\left|f_{s}\pm mf_{i,o}\right|$$

where *m* = 1,2,3, … and *f_i,o_* is one of the distinctive vibration frequencies which are determined by the dimensions of the bearing.

(4) $$f_{i,o}=\frac{n}{2}f_{r}\left[1\pm \frac{bd}{pd}cos\beta \right]$$

where *n* is the number of bearing balls, *f_r_* is the mechanical rotor speed in Hz, *bd* is the ball diameter, *pd* is the bearing pitch diameter, and *β* is the contact angle of the balls on the races.

In this paper, the focus is on the analysis of broken rotor bars.

### 2.3. Machine Learning Algorithms

Machine learning (ML) techniques have been proven effective and dependable for a wide range of applications, including identifying patterns, forecasting, modeling, optimization and data analysis. ML methods can be categorized into supervised, unsupervised, semi-supervised and reinforcement learning [39], depending on the characteristics of the data used and the type of system to be developed.

Supervised learning uses labeled input data to predict an output variable and can be further divided into classification and regression techniques. The standard procedure in supervised learning is to first train a classification model using a labeled dataset that encompasses the relevant classes. Then, the model is tested on another data set, which is unlabeled, in order to evaluate its performance.

The use of machine learning methods is widely used for fault diagnosis of induction machines in literature. It is common to find fault diagnosis solutions based on Artificial Neural Networks (ANN) and Decision Tree (DT) methods, such as Random Forest (RF) and Support Vector Machine (SVM) [40], among others. Renowned methods, such as Random Forest, are continuously being advocated for detecting broken rotor bars [41] or other Decision Trees models for short circuit faults [42]. Several models, including Random Forest and Support Vector Machine, were used to monitor switch open-circuit faults in an inverter-fed induction motor in [28]. An example of another type of solution that employs AI and machine learning is [43], where a genetic algorithm (GA) was presented for bearing fault diagnosis based on information obtained from MCSA.

The research community is devoting significant efforts to real-time and online solutions [10]. In [44], the application of 1-D Convolutional Neural Networks (CNN) is presented, which offers real-time fault detection in a powerful Field-Programmable Gate Array (FPGA) deployment without requiring a feature extraction algorithm using current measurements. In [19], Nearest Neighbour (NN), Linear Discriminant Analysis (LDA), and Support Vector Machine were used to classify rotor faults from vibration data.

Deep Learning techniques can also be applied to classifying faults, such as the use of Deep Convolutional Neural Networks (DCNN) in the case of data obtained from vibration signals for broken rotor bar analysis [21] or for the diagnosis of bearing faults [45].

In [23], a Support Vector Machine was applied to classify thermography images in order to detect short-circuit faults. Meanwhile, in [33], regression models were presented for classifying the severity of eccentricity faults, using data from current harmonics, vibration, rotation speed, and torque, with the aim not only of detecting but also classifying the gravity of the fault. More sophisticated approaches have been taken for classifying nonstationary operations without a dataset for training, such as using an Ensemble framework, Fuzzy Rough Active Learning, and drift detection for detecting broken bars [46].

The availability of training sets can be a challenge in both industrial settings and laboratories, as there may be a limited number of faulty machines for training purposes, and collecting data with multiple faults in a single machine can be difficult [39]. It is also observed that many of the research studies presented in the literature are based on datasets from laboratory tests. It would be interesting to start recording real data from actual environments [47].

The following subsections present a concise overview of the machine learning methods employed in this paper. These methods were chosen due to their high performance, explainability and suitability for implementation on resource-constrained platforms, as is the case at hand.

#### 2.3.1. Decision Trees

**Figure 4:**
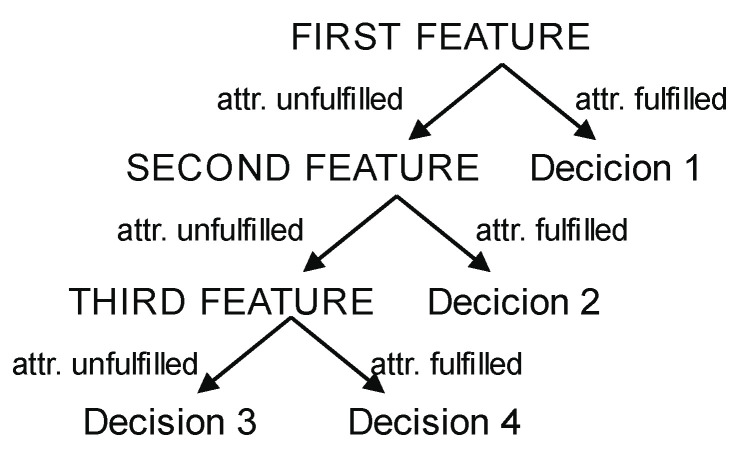
Decision tree diagram.

A decision tree (DT) is a commonly used machine learning method for decision support in data analysis and statistics, with a particular focus on artificial data mining. The goal of DTs is to create a model that predicts the target value based on multiple inputs. Decision Tree methods, therefore, are a widely used model for solving classification and regression problems in the context of supervised learning. The structure of DTs is depicted by branches and leaves, where branches contain the attributes that the function relies on, and leaves contain the function’s value. Other nodes contain attributes that distinguish the decision cases. An illustration of the DT algorithm is presented in Figure 4.

Compared to other decision models, DTs are simple and require only a small amount of data to produce results. They can also be combined with other decision models to increase accuracy. However, undoubtedly, the main feature that makes these methods one of the most widely used is the ease of explanation of the decision process thanks to its tree structure, which inherently allows for reproducing the decision process obtained after training the model. For this reason, decision trees are known as white-box models, as opposed to other models, such as Artificial Neural Networks or Support Vector Machines, which are known as black-box models, since they do not offer such an understandable explanation of the decision process. However, DTs are inherently unstable, and even a small change in input data can result in a significant change in the decision tree structure and potentially lead to inaccurate results.

#### 2.3.2. Random Forest

Decision trees present a dilemma. A deep tree with numerous leaves can result in overfitting as the prediction is based solely on the few features present in its leaves. On the other hand, a shallow tree with few leaves lacks the ability to capture distinctions in the raw data and thus performs poorly.

In contrast, the random forest approach utilizes multiple trees, which are built using random subset attributes. This way, an uncorrelated forest is generated. Then, the prediction is built by taking the average of the predictions made by each individual tree or using other criteria. As a result, it generally exhibits improved predictive accuracy compared to a single decision tree.

#### 2.3.3. Support Vector Machines

**Figure 5:**
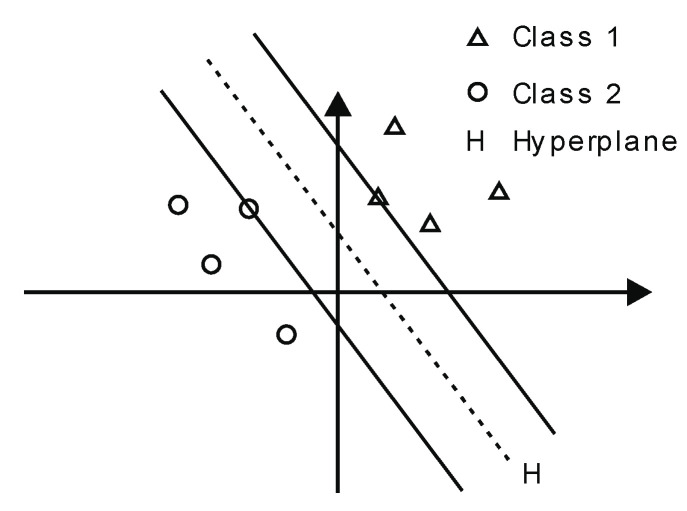
Support Vector Machine.

Intuitively, Support Vector Machines (SVMs) are models that map sample points into a multi-dimensional space, separating them into two distinct classes. This division is achieved by a hyperplane, as demonstrated in Figure 5. The hyperplane is determined by the two closest sample points from each class, referred to as support vectors. These support vectors determine the boundaries of the classes, with the distance between the support vectors of each class, referred to as the margin. Although there are multiple hyperplanes that can effectively classify the samples, it is common practice to select the hyperplane with the greatest margin.

In the figure, two data classes are represented: Class 1 (triangles) and Class 2 (squares). In addition to linear classification, SVMs can deal with non-linear classification using the kernel trick, using polynomial, radial or sigmoid kernels to map the data to a higher dimension where data is separable.

When a multiclassification problem is addressed, let n be the number of classes, a total of $\frac{n\left(n-1\right)}{2}$ binary classifiers must be trained to separate all the pairs of n classes. This way, the predicted class for a new instance is the most voted among all the classifiers built. For that reason, when the number of classes is high, and the dataset is large, SVMs involve an expensive computational cost in terms of time and memory.

#### 2.3.4. Evaluation of Methods

The Confusion Matrix is utilized to assess the effectiveness of various classification techniques by determining the accuracy of a classifier. It is represented as a square matrix, with the dimensions being determined by the number of classes present. In the current scenario, there are two classes: healthy and faulty. The confusion matrix shows the next values:

- True Positives (TP): This occurs when the prediction that an observation belongs to a specific class is accurate, as the observation indeed belongs to that class.
- True Negatives (TN): This occurs when the prediction that an observation does not belong to a specific class is accurate, as the observation indeed does not belong to that class.
- False Positives (FP): This occurs when the prediction that an observation belongs to a specific class is incorrect, as the observation actually does not belong to that class.
- False Negatives (FN): This occurs when the prediction that an observation does not belong to a specific class is incorrect, as the observation actually belongs to that class.

It is necessary to indicate the analysis parameters to measure the performance of the different classification methods. The accuracy is the ratio of true cases to all cases, as shown in (5). The recall is focused on the positive class. It is the ratio of the correct positive predictions to all observations in the positive class, given by (6). The precision is the ratio of the correct positive predictions to all positive predictions, given by (7).

(5) $$\mathrm{Accuracy}=\frac{\mathrm{TP}+\mathrm{TN}}{\mathrm{TP}+\mathrm{TN}+\mathrm{FP}+\mathrm{FN}}$$

(6) $$\mathrm{Recall}=\frac{\mathrm{TP}}{\mathrm{TP}+\mathrm{FN}}$$

(7) $$\mathrm{Precision}=\frac{\mathrm{TP}}{\mathrm{TP}+\mathrm{FP}}$$

F_1_ score is a measure given by (8), being the harmonic mean of precision and recall. F_1_ varies from 0 to 1, with zero as the worst value.

(8) $$F_{1}=2\frac{\mathrm{Precision}\cdot \mathrm{Recall}}{\mathrm{Precision}+\mathrm{Recall}}$$

### 2.4. Industrial Cyber-Physical Systems

A Cyber-Physical System (CPS) combines computational applications with physical devices and is structured as a network of interacting cyber and physical elements [48]. Unlike embedded systems, which primarily focus on computational elements housed in standalone devices, CPSs are primarily designed as a larger network of interacting computational and physical elements [49]. When CPSs are used in industrial environments emergence the Industrial Cyber-Physical Systems (ICPSs) [50] or Cyber-Physical Production Systems (CPPSs) [51], in combination with the widespread use of the Internet of Things (IoT) [52].

Under the subject matter discussed in this paper, the ICPS will consist of various components, but those related to fault diagnosis can be summarized as follows:

- Physical component: it comprises the hardware and all the sensors involved in data acquisition, which is necessary to conduct fault diagnosis.
- Cyber component: it is the intelligent component where data collected from the physical world is analyzed and interpreted. This is where emerging technologies are utilized.

Two different approaches can be taken at this stage: Edge or Fog/Cloud Computing.

#### 2.4.1. Fog/Cloud Computing

In these scenarios, data is collected at the local level and then transmitted to the fog or cloud for further processing. This approach offers high computing power. Researchers often focus on testing or developing new machine learning models using simulation environments such as MATLAB/Simulink [53] or by modifying datasets to include potential failures in order to evaluate the performance of various classification techniques [54]. It is typical for machines to be positioned in a fixed location, although there are exceptions. An example of this is the predictive maintenance of mobile machinery in a mining complex utilizing Bluetooth Low Energy vibration sensors, as presented in [55].

#### 2.4.2. Edge Computing

When the cyber and physical components are situated in close proximity to the electrical machine under examination, this approach can be challenging to implement in industrial settings due to the computational resources required for data processing, necessitating the presence of a nearby personal computer.

In controlled environments, such as laboratories, this setup is prevalent. It typically involves the use of data acquisition cards and specialized software running on a personal computer to provoke faults in an electrical machine and study their effects. In [18], a National Instruments Data Acquisition (NI DAQ) device is utilized to acquire information from a current sensor in order to test various pre-filter techniques for data preparation. In [21], a NI DAQ, a current probe, and a tachometer are utilized to evaluate an induction motor and investigate various machine learning techniques, including neural networks and deep multimodal learning. Reference [17] suggests the utilization of a deep network-based approach to examine the characteristics of thermograms with the aid of a thermal camera. NI DAQ and current sensors are used for short-circuit detection [42]. These methods are suitable for academic settings to conduct experiments and evaluate outcomes to advance new techniques. However, their implementation in industrial environments may prove challenging.

In a limited number of instances, edge computing has been utilized to its fullest potential. As demonstrated in reference [56], a method for detecting bearing faults through vibration analysis utilizing ultra-low power wireless sensors is proposed. These sensors collect and analyze the data and only transmit information pertaining to the operational status of the machine to the rest of the ICPS. This application’s feasibility is made possible through the employment of single-axis accelerometers and the creation of a lightweight neural network classifier.

An edge computing solution for fault diagnosis based on an ICPS architecture implemented over the Arduino platform is presented in the following sections.

## 3. Proposal

This paper presents a rotor bar-breaking fault diagnosis method implemented in an ICPS approach. The subsequent subsections introduce the components of the ICPS. It functions using MCSA data and employs a machine learning-based classification method. Its purpose is to operate at the edge of a platform with limited resources.

### 3.1. ICPS Physical Part

The physical part of the ICPS oversees the data acquisition system. It must collect enough current measurements to extract the components of the power spectrum.

The Arduino platform has been selected because it is looking for an affordable data acquisition system. It includes an analog-to-digital converter (ADC) with a default sampling rate of 9600 Hz. To ensure accurate detection of the faults is required a sampling frequency adheres to the Shannon-Nyquist frequency, thereby avoiding potential issues of aliasing or inaccurate signal reconstruction. Therefore, with the default configuration, it is possible to correctly sense signals up to 4800 Hz. It is also possible to increase the sampling frequency of Arduino, setting the prescaler of the clock. For example, the Arduino Uno is equipped with a microcontroller ATMega328P that includes a clock speed of 16 MHz and a prescale factor of 128 by default. In case of necessity, it is possible to achieve a higher rate of 615 kHz by adjusting the prescale factor and using interrupts. The sampling frequency will be set depending on the physical characteristics of the monitored machine.

**Figure 6:**
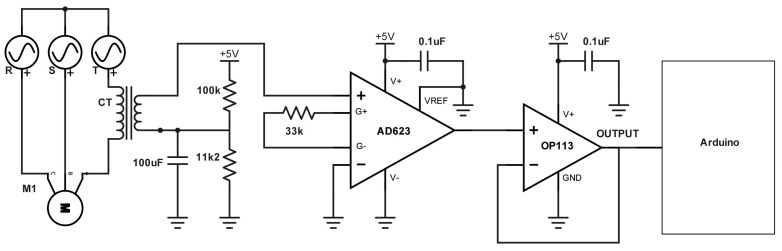
Signal conditioning circuit.

In order to measure the current consumed by induction motors, a non-invasive clamp sensor, model SCT-013-030 by YHDC^®^, was selected. As shown in Figure 6, this sensor is a current transformer with a range of 0–30 A and provides an output voltage range of 0 to 1 V.

Additional hardware has been added to adapt the signal from the sensor to the input of Arduino’s ADC, including an offset and amplification stage by means of AD623 by Analog Devices^®^ and an OP113 operational amplifier was configured in voltage follower to further improve the characteristic curve of the sensor. The final characteristic curve of the sensor is as follows:

(9) $$i\left(t\right)=\frac{30}{4}\left(v\left(t\right)-0.5\right)$$

### 3.2. ICPS Cyber Part

**Figure 7:**
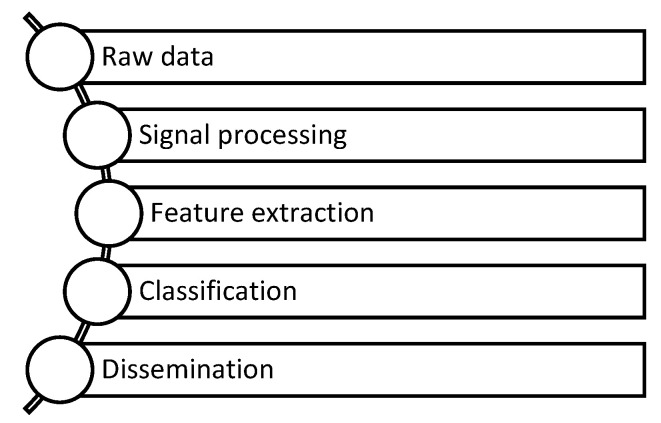
Flowchart of the cyber part.

The cyber component is responsible for processing the data obtained from the sensors, extracting relevant features, classifying them, and communicating the status of the machine to the rest of the ICPS, as depicted in Figure 7.

#### 3.2.1. Signal Processing

Raw data coming from the sensors is a time-based signal. A frequency-based signal is needed to extract the convenient information.

FFT is the chosen method in this paper. So, the signal has to be windowed:

(10) $$i_{R}^{*}\left(t\right)=\left\{\begin{array}{cc} i_{R}^{*}\left(t\right) & for\;t\;\epsilon \;\left[0,\;T\right]\\ 0 & Otherwise \end{array}\right\}$$

And then compute the Fourier Transform:

(11) $$I_{R}^{*}\left(\omega \right)=\overset{\infty }{\underset{-\infty }{\int }}i_{R}^{*}\left(t\right)e^{-j\omega t}dt$$

**Figure 8:**
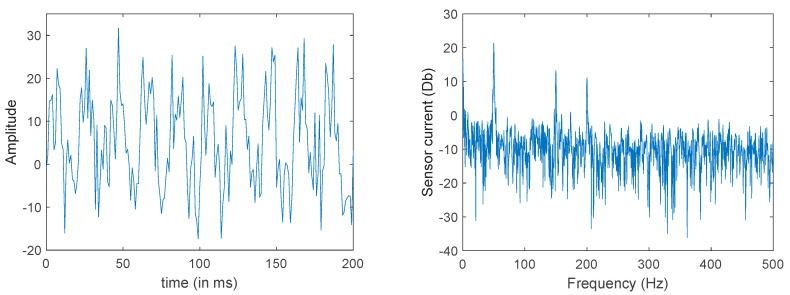
Input signal (**left**) and output signal (**right**) of the signal processing.

The output signal obtained after the FFT calculation of the acquired current signal is presented in Figure 8.

#### 3.2.2. Feature Selection

As stated in previous Section 2.2.2, it is necessary to measure the sidebands of the main frequency to detect broken rotor bars. Taking that information as starting point and given that we will be training the model based on data from a specific motor, but our objective is to design a model that can be used on different motors, it is essential that the definition of the features is extrapolatable to any motor. Hence, the following features have been proposed:

- Difference between the amplitude of the main frequency and the sidebands up to 5th order, in Db. The selection of sidebands is according to Equation (2) in Section 2.2.2. The frequency of these sidebands depends on the characteristics of the motor.
- The relative root mean square (RMS) error of the current signal in comparison with the expected RMS value, its nominal value according to the manufacturer. It is the relative root mean square error (RRMSE). The RMS is calculated in the frequency range covering the above sidebands.

(12) $$I_{rms}=\sqrt{\sum_{j=1}^{n}i_{j,rms}^{2}}$$

(13) $$\mathrm{RRMSE}=\frac{I_{rms,nominal}-I_{rms}}{I_{rms,nominal}}$$

where *i_j,rms_* is the rms current value of the n frequency from the FFT, and *I_rms,nominal_* is the nominal current of the characteristics of the motor.

- The total harmonic distortion (THD) calculated among the range covering the above sidebands:

(14) $$\mathrm{THD}{\left(I\right)}_{f}=\frac{\sqrt{\sum_{j=1}^{n}i_{j,\;rms}^{2}}}{I_{ftal,rms}}$$

where *i_ftal,rms_* is the rms current value of the main frequency.

These two parameters are chosen to provide the model with an idea of the number of harmonics in the signal that could lead to a proper classification.

#### 3.2.3. Classification

It is proposed to apply supervised learning techniques for classifying new observations between healthy or faulty states. The classification is based on Decision Trees (DTs), Random Forest (RF), and Support Vector Machine (SVM), paying special attention to their possible implementation on Arduino.

#### 3.2.4. Dissemination

Upon determining the state of the machine, the system will disseminate the information to the rest of the ICPS that control the system. The mode of communication that is used, either LAN or wireless, will depend on the scenario. For systems that utilize an Arduino Uno, the implementation of a communication shield is a feasible option. However, it may be more efficient to use a UART-LAN/WIFI interface module to handle the communication protocol and allow the Arduino to only transmit the information via the USART port in order to avoid interfering with the normal operation of the microcontroller.

## 4. Case Study

**Figure 9:**
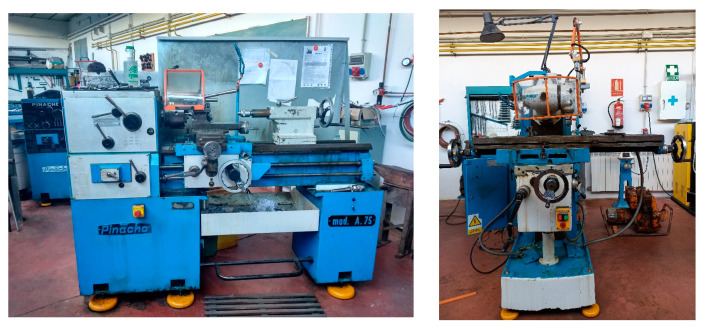
Lathe (**left**) and milling machine (**right**) tested.

The proposed fault detection method based on Arduino boards has been tested on equipment of the Mechanical Engineering Laboratory at the Mining and Industrial Engineering School of Almadén (UCLM-Spain), specifically, a lathe and a milling machine (Figure 9) equipped with a three phases electrical motor AEG AM 100 LZ 4 and a Siemens 1LA3 106-4AA20 respectively, with the characteristics shown in Table 1.

### 4.1. Datasets

In this research, there was no starting dataset available, so the one from the Laboratory of Intelligent Automation of Processes and Systems and Laboratory of Intelligent Control of Electrical Machines, School of Engineering of São Carlos of the University of São Paulo (USP), Brazil [57], is used to train and test the machine learning models.

#### 4.1.1. Raw Data

Their setup consists of a three-phase induction motor coupled with a direct-current machine, which works as a generator connected by a shaft containing a rotary torque wrench, simulating the load torque. In their laboratory they artificially produced rotor broken bars faults and tested the motor from 12.5% to 100% load under a stationary state.

The USP dataset includes 10 tests for each of the 8 operational setups (loads every 5 Nm from 5 Nm to 40 Nm) for 5 states: a healthy motor or a motor with 1, 2, 3 or 4 broken rotor bars. Eleven variables are measured for 19 s:

- 3-phase motor current at 50 kHz sampling frequency.
- 3-phase motor voltage at 50 kHz sampling frequency.
- 5 vibration sensors at 7.6 kHz sampling frequency.

#### 4.1.2. Feature Extraction

The raw data from the motor current was loaded in MATLAB, split into a four seconds test, and processed to calculate the power spectrum by the FFT. By considering the characteristics of the motor tested in the laboratory of USP (with a nominal speed of 1715 rpm and four poles), it is possible to calculate the synchronous speed and the slip. As a result, the relevant sidebands, according to literature alerts of broken rotor bar, are shown in Table 2.

Then, features were calculated following the indications in Section 3.2.2 in the range from 4.5 Hz to 94.5 Hz. This range encompasses all sidebands and mitigates the inaccuracies that arise in the extreme ends of the FFT calculation, particularly when windowing is utilized. The final dataset consists of 12 features and an additional column indicating whether the machine is in a healthy (1) or faulty (−1) state. It comprises 474 cases, equally divided between healthy and faulty cases (237 cases each). The data is organized as a 475 × 13 matrix with 475 rows and 13 columns.

### 4.2. Model Development

**Figure 10:**
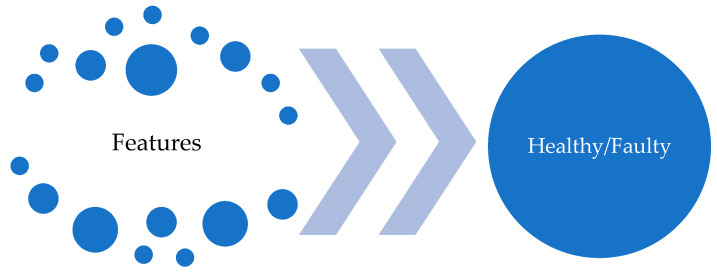
Input and output of the models.

Python programming language and the sci-kit-learn library in the Kaggle environment were used to develop the models, which were later deployed on the Arduino platform. Decision Trees (DT), Random Forest (RF), and Support Vector Machine (SVM) models were trained using the created dataset. The dataset was divided into two parts, with 355 cases utilized for training (which corresponds to 75% of the available data) and 119 cases reserved for testing (the remaining 25%). The selection of training and test set instances has been uniformly performed. The aim of the models is to classify new observations as healthy or faulty depending on the 12 presented features, as Figure 10 shows.

After all the classification algorithms were applied to the test dataset, the classification metrics listed in Section 2.3.4 were derived from the confusion matrix data for each model, shown in Table 3. The results of these metrics are presented in Table 4. It is worth mentioning that the best results obtained with SVM are based on the radial kernel with a regularization parameter, C, equals 1000 and with no maximum leaves defined for DT.

In order to validate these metrics, each model was subjected to a K-fold cross-validation test, yielding consistent results.

It is observed that the DT model provides the highest accuracy, correctly classifying 96.6% of the observation, followed by RF at 87.4% and SVM at 73.9%. With regard to recall, the DT model demonstrates the best performance. Recall measures the ability to correctly predict positive class samples. In certain scenarios, it is imperative to avoid misclassifying any positive class, such as in the case of misclassifying cancer samples. DT achieves a recall score of 96.8%, followed by the RF model at 89.8% and the SVM model at 77.2%. Precision reflects the model’s ability to correctly identify positive predictions, such as avoiding labeling a regular email as spam. In terms of precision, SVM and RF models perform with a score of 71.0% and 85.5%, respectively. The DT model is ahead with a score of 96.8%. Ultimately, the F_1_ score measures the overall performance of the models, and the DT model attains the highest score of 96.8%, followed by RF at 87.6% and SVM at 73.9%.

Throughout the process of training and testing the classification model, we have observed that the RMS value of the signal provides precise results. Notably, an accuracy of nearly 100% was achieved when using the RMS value. However, it should be noted that this may not be applicable to motors with differing characteristics. Therefore, since the goal is to find a versatile model that can be used with different types of motors, an equivalent value, RRMSE, has been used, as was presented in Section 3.2.2.

### 4.3. Implementation of Resource-Constrained Platform

Given that we are working with the resource-constrained platform, Arduino, it is important to optimize the current measurement for better performance and to ensure proper processing of the FFT signal. In order to achieve this, three variables must be optimized: sampling frequency, number of samples, and frequency resolution. The sampling frequency is restricted by the Shannon–Nyquist frequency and by the range of frequencies associated with the fault to be detected. According to the equations described in Section 2.2 and the motor characteristics presented in Table 1, this range can go up to 618.00 Hz (bearing fault), 168.33 Hz (eccentricity) or 80.00 Hz (broken rotor bar).

The number of samples is limited by the memory resources of the Arduino board. The Arduino Uno has 2 KB of RAM and can store a maximum of 128 samples, while the Arduino Mega, with 8 KB of RAM, can store up to 512 samples.

The frequency resolution refers to the spacing between two consecutive frequencies in the power spectrum and, therefore, the frequencies that would be detected. It is calculated as follows:

(15) $$Res_{f}=\frac{f_{sampling}\;\;}{samples}$$

There are two options to improve the resolution: use a smaller *f_sampling_* or increase the number of samples. Both are restricted by the characteristics already commented on. Table 5 shows the achievable resolutions for measuring up to the fifth sideband frequency of the lathe and milling machine for the three types of faults discussed, using either the Arduino Uno (with 128 samples) or the Arduino Mega (with 512 samples).

Based on the results shown in Table 5, it can be observed that it is not possible to carry out the bearing and eccentricity fault diagnosis using the Arduino Uno, as the resolution frequency is quite poor. Using the Arduino Mega, it is possible to carry out the three types of fault diagnosis.

The micromlgen library was utilized to export the classification models from Python to C code. Table 6 summarizes the impact of each model on Arduino. It can be observed that RF has many more code lines than the rest. This is because it calculates 100 DT models and provides the mean value of all of them. SVM has a bigger impact on program space, using 13% more space than the other due to the kernel calculation conducted. Dynamic memory is the same in all the models, as it is related to the FFT calculations. As the results in Table 4, Table 5 and Table 6 show, we can conclude that DT is the best model to be implemented on Arduino.

**Figure 11:**
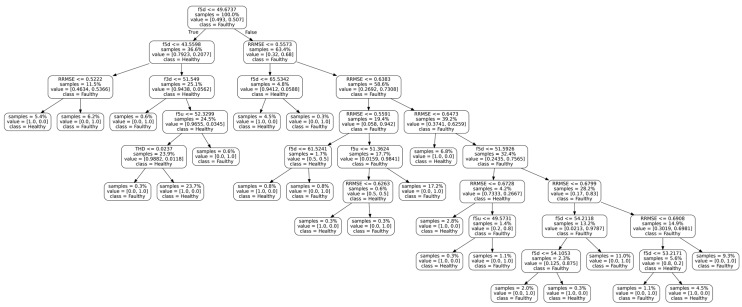
Decision tree model developed.

A graphical representation of the DT model with the decisions made to classify a new observation is shown in Figure 11. It is worth noting the importance that the RRMSE value takes, appearing in 9 out of the 22 decisions made by the decision tree. The theory would suggest that the presence of sidebands is the determining factor, but in this case, it is not as significant.

### 4.4. Real Case

After developing and programming the classification model in the Arduino Mega, a series of tests were conducted on the lathe and milling machine under varying loads to verify their optimal operating conditions. The lathe was tested under no-load conditions, turning 1 mm and 2 mm. The milling machine was tested under no-load conditions, milling 0.1 mm and 1 mm.

Arduino measured the current feeding the motor, calculated the FFT of the signal, extracted and computed the features, and executed the classification model. After that, Arduino concluded that the lathe was faulty, while the milling machine was healthy.

In order to check these results, the process followed by Arduino was replicated in the laboratory. Debug codes were loaded into the Arduino to download the values of the current measurements and the locally calculated FFTs. From there, features were extracted, and the decision tree model was applied.

**Figure 12:**
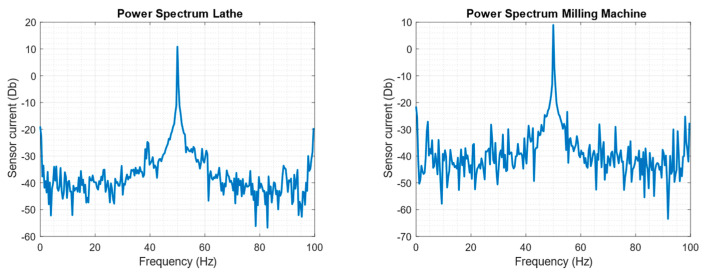
Power spectrum of the lathe (**left**) and the milling machine (**right**) tested.

Figure 12 shows the power spectrum of the lathe and milling machine during tests with a higher load. There are no noticeable peaks in the sidebands of either machine. The windowing effect can be observed at the ends of the figures, leading to inaccurate values in these regions, as mentioned before. Those values are discarded.

The first step to extracting the features is to determine the relevant sidebands of the lathe and the milling machine. The sidebands are shown in Table 7.

For both machines, the features defined in Section 3.2.2 and shown in Table 8 and Table 9 were extracted. Based on these values, it is possible to reproduce the steps followed by the decision tree model. In the case of the lathe, the first leaf compares whether f5d is less than or equal to 49.6737. This first comparison is true, so the process continues to the second leaf. In the second leaf, f5d is compared to 43.5598, and the result is false. The next leaf compares whether f3d is less than or equal to 51.549. In this case, the result is true, so the model classifies the machine as faulty. This result was completely unexpected since it was thought that the machine was in proper condition. The same result is achieved using data from other tests in which the load is varied.

In the milling machine classification, the process begins similarly to the previous case by comparing if f5d is less than or equal to 49.6737. The comparison result is negative, so we proceed to the leaf where it is compared if RRMSE is less than or equal to 0.5573, which is true. We then continue to compare if f5d is less than or equal to 65.5342, which is correct, and therefore it is determined that the machine is healthy.

Following the successful implementation of the models on the Arduino 2560, it has been demonstrated that platforms with limited resources, such as Arduino, can execute machine learning models to undertake fault diagnosis tasks.

## 5. Conclusions

In this paper, an edge computing approach for fault diagnosis is proposed. The system has been designed as an Integrated Cyber-Physical System (ICPS) embracing the physical and the cyber world. Both parts have been developed using the Arduino platform, specifically the Arduino Mega board.

A classification method was developed by utilizing a publicly available dataset containing information on a three-phase inductive motor under both healthy and broken bar conditions. The purpose was to create a model that could be applied to other three-phase induction motors with varying features. For this, novel features were defined based on well-known MCSA sidebands. However, during the development of the model, it was discovered that the sidebands are not as crucial to the classifiers as previously believed.

The research has shown that machine learning models, including Decision Trees, Random Forests, and Support Vector Machines, can be executed satisfactorily on resource-constrained platforms such as the Arduino Mega. However, due to memory limitations, the amount of data that can be stored is limited, which in turn limits the features that can be extracted using the Fast Fourier Transform.

The process of feature extraction, classification, and model training and testing has been presented and implemented in Arduino Mega, testing two electric motors in the laboratories of the Industrial and Mining School of Almadén.

The proposed solution is cost-effective for small to medium-sized enterprises, enabling them to embrace the digitalization era and reap its benefits.

Further research is aimed at incorporating more complex classifiers that draw information from multiple sensors, not only to detect faults but also to make prognoses.

## Figures and Tables

**Table 1:** Characteristics of the Electrical Motor tested.

Description	Lathe	Milling Machine
Manufacture	AEG	Siemens
Power	4 kW	2 kW
Frequency	50 Hz	50 Hz
Voltage	230/400 V	230/400 V
Current	13.94/8.04 A	6.97/4.03 A
Number of revolutions	1420 rpm	1410
Number of poles	4	4

**Table 2:** Sidebands of interest for developing the classification model.

	K = 1	K = 2	K = 3	K = 4	K = 5
Upper	65.67	71.33	77.00	82.67	88.33
Lower	54.33	48.67	43.00	37.33	31.67

**Table 3:** Comparison of classifier performance using a confusion matrix.

	DT	RF	SVM
TP	60	53	44
TN	55	51	44
FP	2	9	18
FN	2	6	13

**Table 4:** Comparison of classifier performance using evaluation metrics.

	DT	RF	SVM
Accuracy	0.958	0.866	0.714
Recall	0.952	0.895	0.755
Precision	0.967	0.836	0.656
F_1_	0.959	0.864	0.702

**Table 5:** Resolution frequency for each type of fault using the Arduino Uno or the Arduino Mega for the lathe (L) and milling machine (M).

	Max Frequency	Sampling Freq.	Num. of Samples	Resolution
Bearing fault	618.00 Hz (L)	1236.00	128	9.66
	614.00 Hz (M)		512	2.41
Eccentricity	168.33 Hz (L)	336.67	128	2.63
	167.5 Hz (M)		512	0.66
Broken rotor bar	73.61 Hz (L)	147.22	128	1.15
	80.00 Hz (M)		512	0.29

**Table 6:** Impact of the models on Arduino sketch.

	Code Lines	Program Space	Dynamic Memory
DT	133	6924 bytes (2%)	4341 bytes (52%)
RF	26,571	6924 bytes (2%)	4341 bytes (52%)
SVM	224	40,294 bytes (15%)	4341 bytes (52%)

**Table 7:** Sidebands of interest for lathe and milling machine testing.

		K = 1	K = 2	K = 3	K = 4	K = 5
Lathe	Upper	55.33	60.67	66.00	71.33	76.67
	Lower	44.67	39.33	34.00	28.67	23.33
Milling machine	Upper	56.00	62.00	68.00	74.00	80.00
	Lower	44.00	38.00	32.00	26.00	20.00

**Table 8:** Extracted features for lathe classification.

Feature	Value	Feature	Value	Feature	Value
f1d	40.2260	f3d	45.8927	f5d	45.9219
f1u	38.1941	f3u	49.5721	f5u	46.4160
f2d	35.5643	f4d	51.9447	THD	1.0342
f2u	38.8301	f4u	47.1394	RRMSE	0.6803

**Table 9:** Extracted features for milling machine classification.

Feature	Value	Feature	Value	Feature	Value
f1d	45.8208	f3d	40.9054	f5d	52.3244
f1u	42.2207	f3u	43.6715	f5u	49.1608
f2d	47.9009	f4d	46.8002	THD	1.0255
f2u	49.6617	f4u	48.6237	RRMSE	0.4917

## Data Availability

Not applicable.
